# Determination of ivermectin in plasma and whole blood using LC-MS/MS

**DOI:** 10.12688/wellcomeopenres.20613.2

**Published:** 2024-08-05

**Authors:** Natpapat Kaewkhao, Warunee Hanpithakpong, Joel Tarning, Daniel Blessborn

**Affiliations:** 1 Mahidol Oxford Tropical Medicine Research Unit, Faculty of Tropical Medicine, Mahidol University, Bangkok, 10400, Thailand; 2 Centre for Tropical Medicine & Global Health, Nuffield Department of Clinical Medicine, University of Oxford, Oxford, UK

**Keywords:** Development, Human blood, Ivermectin, LC-MS/MS, Malaria, Validation

## Abstract

**Background:**

Ivermectin is a widely used drug for the treatment of helminthiasis and filariasis worldwide, and it has also shown promise for malaria elimination through its potent mosquito-lethal activity. The objective of this study was to develop and validate a high-throughput and sensitive method to quantify ivermectin in plasma and whole blood samples, using automated sample extraction followed by liquid chromatography-tandem mass spectrometry (LC-MS/MS).

**Methods:**

Phospholipids were removed in patient whole blood (100 µl) and plasma (100 µl) samples using a 96-well plate Hybrid-solid phase extraction technique. Ivermectin and its isotope-labelled internal standard (ivermectin-D2) were separated on an Agilent Poroshell 120 EC-C18 50mm × 3.0mm I.D. 2.7µm, using a mobile phase of acetonitrile: ammonium formate 2 mM containing 0.5% formic acid (90: 10, v/v). Detection was performed using a triple quadrupole mass spectrometer in the positive ionization mode.

**Results:**

The method was validated in the concentration range 0.970 - 384 ng/ml in both plasma and whole blood matrices. Intra- and inter-batch precisions during the validation were below 15%. There was no carryover or matrix effects detected. Ivermectin is a stable compound and results showed no degradation in the different stability tests.

**Conclusions:**

The validated method proved to have high sensitivity and precision, good selectivity and to be suitable for clinical application or laboratory quantification of ivermectin in plasma or whole blood samples.

## Introduction

**Figure 1.**
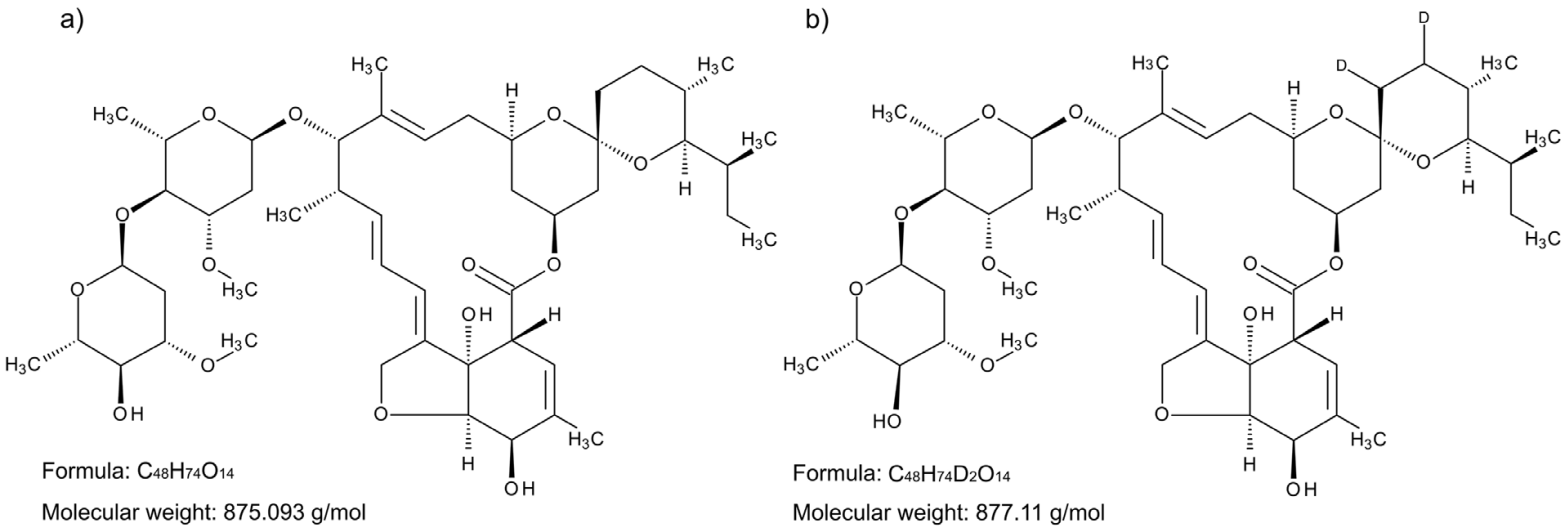
Molecular structure of **a**) ivermectin, and **b**) isotope-labelled ivermectin-D2.

Ivermectin is a macrocyclic lactone anthelmintic compound derived from *Streptomyces avermitilis*. Its chemical structure ( Figure 1) is a mixture of mostly (>90%) avermectin H2B1a (22, 23-dihydroavermectin B1a) with a minor contribution (<10%) of avermectin H2B1b (22, 23-dihydroavermectin B1b). Ivermectin was discovered in 1975, and it is used to treat a range of parasitic diseases in both humans and animals ( Campbell, 1992; Laing *et al.*, 2017; Omura, 2008; Tambo *et al.*, 2015; Van Voorhis *et al.*, 2015; Warrell & Gilles, 2002). It is widely recognized as a crucial drug for the treatment of helminthiasis globally, particularly valued for its effectiveness against filarial infections ( WHO, 2023). The Mectizan ^®^ ivermectin donation program reaches more than 300 million people in affected areas annually, with more than 4.4 billion treatments donated since 1987. In animals, ivermectin is used for the prevention and treatment of heartworms and acariasis ( Sutherland, 1990). In humans, it is used to treat various parasitic diseases and worm infections, such as ticks ( Sheele *et al.*, 2014), head lice ( Glaziou *et al.*, 1994), scabies ( Khan & Yasmin, 2016), river blindness ( Walker *et al.*, 2017), strongyloidiasis ( Datry *et al.*, 1994; Hailu *et al.*, 2020), trichuriasis ( Wimmersberger *et al.*, 2018), ascariasis ( Belizario *et al.*, 2003), lymphatic filariasis ( Cupp, 1992; Onwujekwe *et al.*, 2001; Osei-Atweneboana *et al.*, 2011), onchocerciasis ( Cupp, 1992; Onwujekwe *et al.*, 2001; Osei-Atweneboana *et al.*, 2011), and additional helminths ( Smits, 2009). In addition to treating parasitic and worm infections, ivermectin has been shown to also have paralyzing and killing effects on *Anopheles* mosquito after ingestion with a blood meal ( Chaccour *et al.*, 2010; Cupp *et al.*, 2011; Ottesen *et al.*, 1997; Shoop *et al.*, 1995). In malaria, ivermectin is currently investigated in drug combination therapy with artemisinin-based combination therapies where artemisinin and its partner drug kill the blood stage parasites and ivermectin paralyze and kill mosquitoes that feed on the patient, thereby more effectively stopping the transmission of malaria ( Ouedraogo *et al.*, 2015; Tambo *et al.*, 2015). Ivermectin primarily targets the glutamate-gated chloride channel in *Anopheles* mosquitoes affecting its motility, feeding, and reproduction ( Chaccour *et al.*, 2010; Cupp *et al.*, 2011; Meyers *et al.*, 2015; Ottesen *et al.*, 1997). Ivermectin can also inhibit the sporogony of *P. vivax* malaria parasites in mosquitoes by inhibiting the development of oocysts ( Kobylinski *et al.*, 2017). It is critical to develop sensitive and accurate methods to measure ivermectin in different biological matrices, such as plasma and whole blood, to enable the quantification of concentration-response relationships for these different indications.

Several ivermectin liquid chromatography (LC) quantification methods have been published for animal veterinary use and to confirm drug residues in animal products, river sediment, soil and crops ( Ali *et al.*, 2000; Dahiya *et al.*, 2010; Durden, 2007; Hoyos *et al.*, 2017; Ortiz *et al.*, 2017; Yoshii *et al.*, 2000). Many of these methods use fluorescence detection ( Canton *et al.*, 2018; Danaher *et al.*, 2001; Kitzman *et al.*, 2006; Na-Bangchang *et al.*, 2006; Prieto *et al.*, 2003; Wang *et al.*, 2009), but a large volume of sample (~1000 µl) is needed to achieve sensitive quantification of ivermectin. Ivermectin is distributed heavily into human tissue and skin resulting in low plasma/blood concentrations ( Chaccour *et al.*, 2017; Chhonker *et al.*, 2018; Duthaler *et al.*, 2019; Munoz *et al.*, 2018; Schulz *et al.*, 2018), and only a limited number of assays are developed using mass spectrometry (MS) detection for sensitive and accurate quantification of ivermectin in small volume samples ( Chhonker *et al.*, 2018; Duthaler *et al.*, 2019; Schulz *et al.*, 2018). These methods present different strengths and weaknesses ( Table 1). An assay utilized protein precipitation, which is a fast sample clean-up technique resulting in great sensitivity (0.5 ng/ml) and high recovery (80–120%), but residual proteins might still be present in the sample that can lead to reduced LC-column efficiency and MS matrix effects ( Duthaler *et al.*, 2019). One method used solid phase extraction (SPE), which is a more selective sample extraction technique resulting in high sensitivity (0.1 ng/ml) and a clean sample, optimal for MS detection ( Chhonker *et al.*, 2018). However, this published method used sub-optimal LC separation conditions resulting in a relatively long analysis run time of 10 min per injection, which makes the method less suitable for implementation in a high-throughput laboratory analyzing large sample batches from pharmacokinetic studies ( Chhonker *et al.*, 2018). Here, we present an optimized method with a faster sample extraction process for the quantification of ivermectin in human whole blood or plasma using an effective automated sample extraction, followed by LC separation combined with a sensitive tandem mass spectrometry quantification (MS/MS). The method described here was validated in accordance to the Guidance for Industry from the US Food and Drug Administration (FDA) ( FDA, 2018) and the European Medicines Agency (EMA) ( EMA, 2012). The developed method was implemented in a high-throughput bioanalysis laboratory and applied in the analysis of clinical trial samples.

**Table 1.** Available LC-MS/MS assays to quantify ivermectin in human and non-human samples.

Sample source	Matrix / Sample volume	Extraction method	Column Type	Mobile Phase	Elution technique	Extraction recovery	Injection volume	Analysis run time	Linear range	Reference
Human	WB, Plasma / 100 µl	Phospholipid removal plate Hybrid SPE ^®^ Plus	Poroshell 120 EC-C18, 50×3.0mm, 2.7µm	MPA = ACN: NH _4_FA (2 mM) + 0.5%FA (90:10, v/v) MPB = MeOH: ACN (75:25, v/v).	Gradient	WB = 96-101% Plasma = 108-117%	5 µl	5 min	WB, Plasma 0.970–384 ng/ml	Developed method
Human	WB, Plasma / 50 µl	PP	A Kinetex C8, 50×2.1mm, 2.6µm	MPA = NH _4_FA (20 mM) + 0.1%FA (pH 3.5) MPB = MeOH + 0.1%FA	Gradient	WB = 97.9% P = 108.8%	10 µl	2.5 min	WB = 0.5–100 ng/ml Plasma = 0.5–250 ng/ml	( Duthaler *et al*., 2019)
Human	Plasma / 100 µl	SPE	Luna C8, 30×2.0mm, 3µm	MPA = NH _4_Ac (0.4 mM) + 0.1%FA MPB = ACN + 0.1%FA	Gradient	81%	N/A	4 min	2–200 ng/ml	( Schulz *et al*., 2018)
Human / Mouse / monkey	Plasma / 100 µl	SPE	ACE C18, 50×3mm, 3µm	MPA = Water + 0.1%AA MPB = MeOH: ACN (1:1, v/v)	Isocratic	>80%	10 µl	10 min	0.1–1000 ng/ml	( Chhonker *et al*., 2018)
Dog	Plasma / 500 µl	PP	Agilent Zorbax RRHD Eclipse Plus C18, 2.1×50mm, 1.8µm	MPA = NH _4_FA (5 mM) +0.1%FA MPB = MeOH	Gradient	>80%	10 µl	15 min	0.5–20 ng/ml	( Morbidelli *et al*., 2018)

AA, Acetic acid; ACN, Acetonitrile; FA, Formic acid; MeOH, Methanol; MPA, Mobile phase A; MPB, Mobile phase B; NH _4_Ac, Ammonium acetate; NH _4_FA, Ammonium formate; PP, Protein precipitation; SPE, Solid-phase extraction; WB, Whole blood; v/v, volume by volume percentage.

## Methods

### Ethical conduct of research

Ethical approval for collecting blank human volunteer blood in this study, was granted from the ethics committee of the Faculty of Tropical Medicine, Mahidol University, Bangkok, Thailand, obtained on 31 March 2017 (Approval number MUTM 2017-014-01). Data collection started on 15 May 2017. Informed consent was obtained in writing. Blood donors who showed interest in participating in the study signed written consent forms and underwent screening assessments, including vital sign measurements and routine laboratory blood tests. The donated blood tubes were blinded at the Healthy Volunteer Ward and then transferred to the clinical laboratory.

### Chemicals and reagents

Ivermectin reference standard (purity >94%) was obtained from Sigma-Aldrich (Missouri, United States, I8898). The stable isotope-labelled internal standard, ivermectin-D2 (purity >99.6%), was obtained from Clearsynth (Mumbai, India, CS-O-06696). MS grade water (9831-03), acetonitrile (9829-03) and methanol (9830-03) were obtained from JT Baker (Phillipsburg, USA). MS grade formic acid (56302) and ammonium formate (55674) were obtained from Fluka (Sigma-Aldrich, MO, USA). Blank plasma with citrate phosphate dextrose (CPD) were obtained from the Thai Red Cross, Bangkok, Thailand. Blank whole blood/plasma with sodium-heparin (Leo Pharma, Ballerup, DK, 010040-04) and EDTA (Teklab, Co Dur, UK, K900PP) were collected from healthy volunteers at the Faculty of Tropical Medicine, Mahidol University, Thailand.

### Calibration standards and quality control samples

Stock solutions (1 mg/ml) of ivermectin and ivermectin-D2 were dissolved in methanol. Working solutions were prepared by diluting stock solutions in methanol. Working solutions were used to spike blank CPD whole blood/plasma for calibration standards and quality control samples. The final volume of working solution in blood/plasma was kept below 5% in all samples. Calibration standards were prepared in the range of 0.970-384 ng/ml, where the lowest and highest concentration in the calibration range represent the lower limit of quantification (LLOQ) and upper limit of quantification (ULOQ). Quality control (QC) samples were spiked at three concentration levels, representing a low (3.39 ng/ml), median (33.4 ng/ml), and high (308 ng/ml) concentration in the calibration range. Over-curve samples were prepared at about 3×ULOQ and diluted 10-times with blank plasma before analyzed.

### Sample preparation

An automated liquid handler platform (Freedom Evo 200, TECAN, Mannedorf, Switzerland) was used for the sample preparation process. On each day of analysis, frozen pre-spiked samples, of whole blood and plasma were thawed at room temperature and mixed thoroughly, and then centrifuged at 20°C for 5 minutes at 3000×g. After the 24 hours at -80 °C, the spiked whole blood sample showed complete hemolysis and could be aliquoted similarly to the plasma sample. Whole blood/plasma samples (100 µl) were manually aliquoted into a 96-wellplate and the liquid handler added 450 µl acetonitrile:water (90:10, v/v) containing stable isotope-labelled internal standard (80 ng/ml of ivermectin-D2). The samples were mixed on a Mixmate (Eppendorf, Hamburg, Germany) (1000 rpm, 10 min) and centrifuged (1100×g, 5 min). Using the liquid handler, 380 µl of extracted sample supernatant was loaded onto a Hybrid SPE ^®^ Plus Full Skirt 96-wellplate (575659-U, Supelco, Sigma-Aldrich, MO, USA) and vacuum (3-4 inch Hg) was applied for 30 seconds until the wells became dry. The collected eluate was diluted with 100 µl of mobile phase consisting of acetonitrile:ammonium formate 2 mM with 0.5% formic acid (90:10, v/v).

### LC-MS/MS

The LC system was a Dionex Ultimate 3000 system consisting of a binary LC pump, a vacuum degasser (LPG-3400RS), a temperature-controlled micro-well plate autosampler set at 10°C (WSP-3000TBRS) and a temperature-controlled column compartment set at 40°C (TCC-3000RS) (Thermo Scientific, MA, USA). Data acquisition and processing were performed using Analyst 1.7 ( Sciex, MA, USA). The analytes were separated on a Poroshell 120 EC-C18 50 mm × 3.0 mm I.D. 2.7 µm (Agilent technologies, CA, USA, 699975-302), with a pre-column C18 AJO-7596 4 mm×2 mm, I.D. 3.0 µm (Phenomenex, Torrance, California, USA), at a flow rate of 500 µl/min. The mobile phase consisted of (A) acetonitrile:ammonium formate 2 mM with 0.5% formic acid (90:10, v/v) and (B) methanol:acetonitrile (75:25, v/v). The gradient started with 100% of mobile phase A for 2.10 min (0-2.10 min), followed by immediate switch to 100% of mobile phase B for 1 min (2.10-3.10 min), after which the mobile phase was immediately returned to 100% A and equilibrated until the next sample injection (3.10-5 min). The total runtime was 5 min per sample and the injection volume was 5 µl.

An API 5000 triple quadrupole mass spectrometer ( Sciex, MA, USA) with a TurboV ionization source interface, operating in the positive ion mode, was used for the MS/MS analysis. Ion spray voltage was set to 5500 V, with a drying temperature at 450°C. The curtain gas (CUR) was 30 psi, and the nebulizer (GS1) and auxiliary (GS2) gases were 45 psi and 50 psi, respectively. All collision energy was set to 35 V. Quantification was performed using selected reaction monitoring (SRM) for the specific transitions of ivermectin (m/z 892.5→307.1) and for ivermectin-D2 (m/z 894.5→309.1).

### Method validation

Method validation was performed in accordance with the Guidance for Industry from the US FDA ( FDA, 2018) and the EMA ( EMA, 2012). The calibration curves were analyzed as duplicate samples of each concentration (0, 0.970, 3.39, 11.9, 41.6, 146, and 384 ng/ml). Non-weighted and weighted (1/x and 1/x ^2^) linear regression models were evaluated for the best performing calibration curve model. The selected model was chosen based on the accuracy of back-calculated concentrations of the calibration curves and quality control (QC) samples from four independent validation runs ( Singtoroj *et al.*, 2006). Intra-assay and inter-assay accuracy (mean relative error, %) and precision (%CV) were determined by analyzing five replicates of the LLOQ sample, ULOQ sample, QC samples and over curve sample. A single factor ANOVA was used for precision (%CV) calculation.

Recovery and quantitative matrix effects using the simplified approach described by Matuszewski *et al.*, ( Matuszewski *et al.*, 2003) were evaluated using three sets of samples: Set A: Analyte and internal standard in matrix free “neat” solution, Set B: Extracted blank plasma and/or blood with post-extract addition of analyte and internal standard (post-spiked), and Set C: Blood and/or plasma with added analyte (pre-spiked) and internal standard before extraction. Matrix effect was calculated as (B/A); absolute recovery as (C/B), and recovery as process efficiency (C/A). Recovery was determined by five replicates of extracted QC samples at low (QC1) and high (QC3) concentration level. Quantitative matrix effects (B/A) were evaluated using samples from each of the six blood donors. Evaluation of matrix effect from different anticoagulants used blood collected from a single donor. The calculated (B/A) should be within 0.85–1.15 to be considered free from matrix effects.

Selectivity and qualitative matrix effects were performed by injecting extracts from six different donors and possible co-administered drug during a “normal run” and during post-column infusion. Lumefantrine (100 ng/ml as neat solution) was selected as it is the most commonly used antimalarial partner drug, and it is a highly lipophilic drug with a high protein binding, similar to ivermectin. Primaquine, dihydroartemisinin and piperaquine, (100 ng/ml) were selected due to the coadministration of these compounds with ivermectin in the clinical trial analyzed ( Kobylinski *et al.*, 2020). Post-column infusion solution mix was ivermectin (20 ng/ml) and its stable isotope-labelled internal standards (ivermectin-D2; 20 ng/ml). Possible effects from different anticoagulants, including EDTA hemolyzed plasma, were also investigated by injecting extracts from a single blood source collected in different anticoagulants (EDTA, Heparin and CPD) to evaluate ion suppression or enhancement. A suppression or enhancement of the signal during infusion and at the retention time of ivermectin would indicate an interference. For selectivity, a signal less than 20% of LLOQ at the retention time of the analyte is considered free of interferences ( Ye & Pao, 2015).

Carryover was investigated by injecting five replicates of ULOQ samples followed by three blank samples. Carryover was confirmed if blank sample showed a signal above 20% of LLOQ.

Short term stability was evaluated at two QC levels (low and high). Whole blood and plasma samples were kept in specific conditions e.g. freezer (-80°C), refrigerator (4°C) and ambient temperature (22°C) for 4 hrs, 24 hrs and 48 hrs. Other stability conditions were sample precipitation (stored at 4°C for 24 hrs) and LC autosampler at 10°C.

Long term stability samples stored at -80°C was also evaluated using freshly spiked calibration curve.

### Clinical applicability and incurred sample reanalysis (ISR)

The validated whole blood and plasma methods were applied to a clinical study to quantify ivermectin concentrations in samples from malaria patients ( Kobylinski *et al.*, 2020). The reliability of the methods was evaluated by incurred sample reanalysis ( FDA, 2018; Fluhler *et al.*, 2014). Samples were selected to reflect the overall range of observed clinical drug concentrations (i.e., peak concentrations as well as samples in the elimination phase of the pharmacokinetic profile). For ISR, the difference between the initial and measured concentration during the repeat analysis should be within 20% to be accepted. If the difference in concentration exceeds 20%, the repeat sample is rejected. At least 67% of the ISR set has to pass this criterion or else the method is not considered reproducible.

## Results & discussion

### Method development

Liquid chromatography was optimized by evaluating four reverse phase columns; Hypersil gold C18 100×2.1mm 5µm (Thermo Scientific, MA, USA, 25005-102130), Gemini C18 50×2.0mm 5µm (Phenomenex, CA, USA, 00B-4435-B0), Zorbax SB-CN 50×4.6mm 3.5µm (835975-905) and Poroshell EC C18 50×3 mm 2.7 µm (699975-302) (Agilent technologies, CA, USA). The mobile phase evaluation was performed using organic solvents and buffers, consisting of ammonium formate and ammonium acetate with and without additives. The retention and separation of ivermectin was initially evaluated for the four different columns using an isocratic mobile phase of either methanol or acetonitrile (50–90%). A symmetrical peak shape of ivermectin was achieved with an acidified high organic content (90% methanol or 80% acetonitrile) (Figure S1 to Figure S9 and Table S1, which can be found as *Extended data* ( Kaewkhao, 2023b)). There was no apparent difference in ivermectin chromatography peak shape or signal intensity when using buffers containing 0.1% or 0.5% of formic acid (Figure S8 and Figure S9 ( Kaewkhao, 2023b)). However, separation of ivermectin on the Hypersil gold (Figure S2 and Figure S3 ( Kaewkhao, 2023b)) and Poroshell (Figure S6 and Figure S7 ( Kaewkhao, 2023b)) columns resulted in a higher signal intensity when using ammonium formate (10 mM) with 0.5% formic acid compared to ammonium acetate (10 mM) with 0.5% acetic acid with nearly identical ivermectin retention between the two buffers. Finally, ivermectin had the desired retention and separation on Poroshell 120 EC C18 50×3 mm 2.7µm column using an isocratic mobile phase (A) consisting of acetonitrile:2mM ammonium formate containing formic acid 0.5% (90:10, v/v) (Figure S9 ( Kaewkhao, 2023b)). A mobile phase (B) consisting of methanol:acetonitrile (75:25, v/v), was used as a washout solvent to remove any retained compounds that could potentially cause matrix effects and decrease column performance over time.

Under these conditions, the formation of ivermectin-ammonia adducts ([M+NH4] ^+^) in the positive electrospray ionization mode was unavoidable and was the most intense and reliable ion formation. Therefore, the ivermectin-ammonium adduct was used for quantification in this method. Several other LC-MS/MS method publications also use the ammonium ion adduct for ivermectin ionization ( Durden, 2007; Duthaler *et al.*, 2019; Hoyos *et al.*, 2017; Schulz *et al.*, 2018).

Ivermectin’s ability to stay in solution with different organic content (10–90% of acetonitrile and methanol) were evaluated. The result demonstrated that a minimum of 40% organic content was needed to prevent sample loss due to insolubility (Figure S13 ( Kaewkhao, 2023b)). Ivermectin is a lipophilic compound from its aromatic ring structure, which makes it soluble in hydrophobic solvents such as alcohol. Therefore, when preparing standards and working solutions, it is very important to select an appropriate dilution solution to avoid precipitation. The highest ivermectin extraction recovery was achieved using acetonitrile (90–99%) as precipitation solution with or without acid, and the precipitation solution composition for the final method was 90% acetonitrile in water.

The upper limit of the ivermectin calibration curve was set based on a very minor isotope mass contribution to the internal standard, ivermectin-D2. This could affect the accuracy of ivermectin quantification at high concentrations as an increasing concentration of ivermectin would result in an increasing contribution to the ivermectin-D2 signal, resulting in inaccurate quantification data of ivermectin. According to the FDA and EMA criteria’s, isotope-labelled internal standard interference should not exceed 5% of the average internal standard responses ( EMA, 2012; FDA, 2018). The concentration of ivermectin-D2 solution (80 ng/ml) was set high enough to avoid any substantial effects from ivermectin mass contribution. The mass contribution from ivermectin at the selected upper limit of quantification produced an interference to the ivermectin-D2 trace of less than 3% compared with the normal peak height of ivermectin-D2 and was therefore within acceptable criteria. This contribution is so small that it does not lead to a biased quantification. Hence, the selected calibration curve ranged from 0.97 ng/ml to 384 ng/ml. Furthermore, the upper limit of the quantification was approximately 6-fold higher than the average peak plasma concentrations measured in human healthy Thai volunteers after a single oral standard dose of ivermectin (400 µg/kg) ( Kobylinski *et al.*, 2020) and approximately 3.7-fold higher than that seen after three days of oral high-dose ivermectin (600 µg/kg/day) in adult patients with uncomplicated malaria in western Kenya ( Smit *et al.*, 2019). It has been previously reported that ivermectin concentrations are up to 40% higher in plasma compared with whole blood ( Duthaler *et al.*, 2019). This could be explained by the high binding of ivermectin to human serum albumin ( Okonkwo *et al.*, 1993). However, to keep our procedure simple, the calibration range was kept the same for both plasma and whole blood assay.

### Method validation

Linearity, accuracy and precision were analyzed over four days of validation. The ivermectin whole blood/plasma calibration curve was validated in the range of 0.970-384 ng/ml, and the best performing calibration model for quantification was linear regression with 1/x ^2^-weighting. The correlation coefficient (r ^2^) was > 0.99.

**Figure 2.**
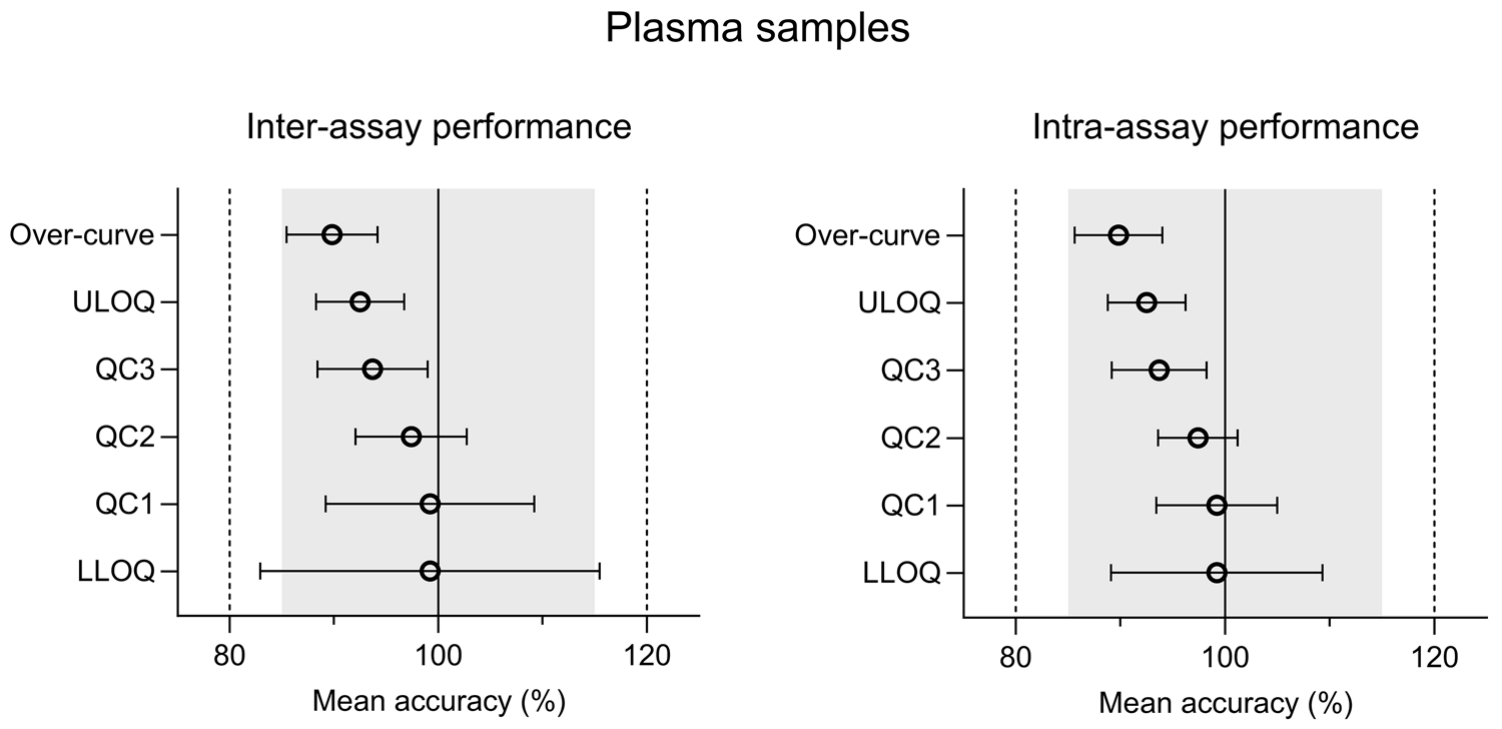
Inter-assay and intra-assay performance of CPD plasma validation samples; quality control (QC), lower limit of quantification (LLOQ), and upper limit of quantification (ULOQ). Inter-assay and intra-assay accuracy was calculated using a modified ANOVA.

**Figure 3.**
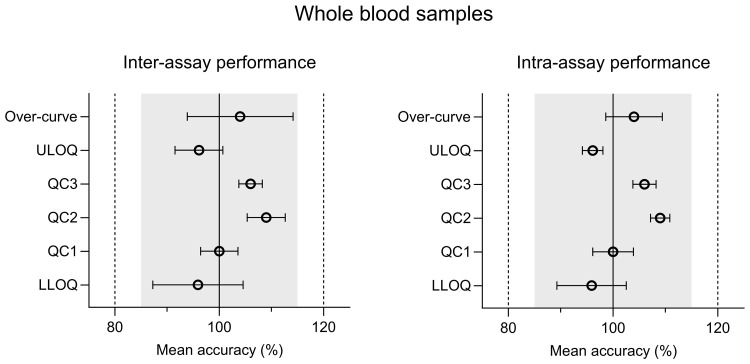
Inter-assay and intra-assay performance of CPD whole blood validation samples; quality control (QC), lower limit of quantification (LLOQ), and upper limit of quantification (ULOQ). Inter-assay and intra-assay accuracy was calculated using a modified ANOVA.

The intra-assay and inter-assay analysis of ivermectin resulted in accuracy values of 89.8%–99.2% for plasma and 95.9%–109% for whole blood. The inter-assay and intra-assay precision (%CV) varied from 3.91% to 16.4% (at LLOQ) for plasma and 1.70% to 9.76% (at LLOQ) for whole blood ( Figure 2 and Figure 3) ( Kaewkhao, 2023a). Both accuracy and precision were within the allowed regulatory criteria of less than 15% deviation (20% at LLOQ). The alternative anti-coagulants (EDTA and Heparin) in plasma and whole blood sample all met the acceptance criteria (Table S2 ( Kaewkhao, 2023b)).

**Figure 4.**
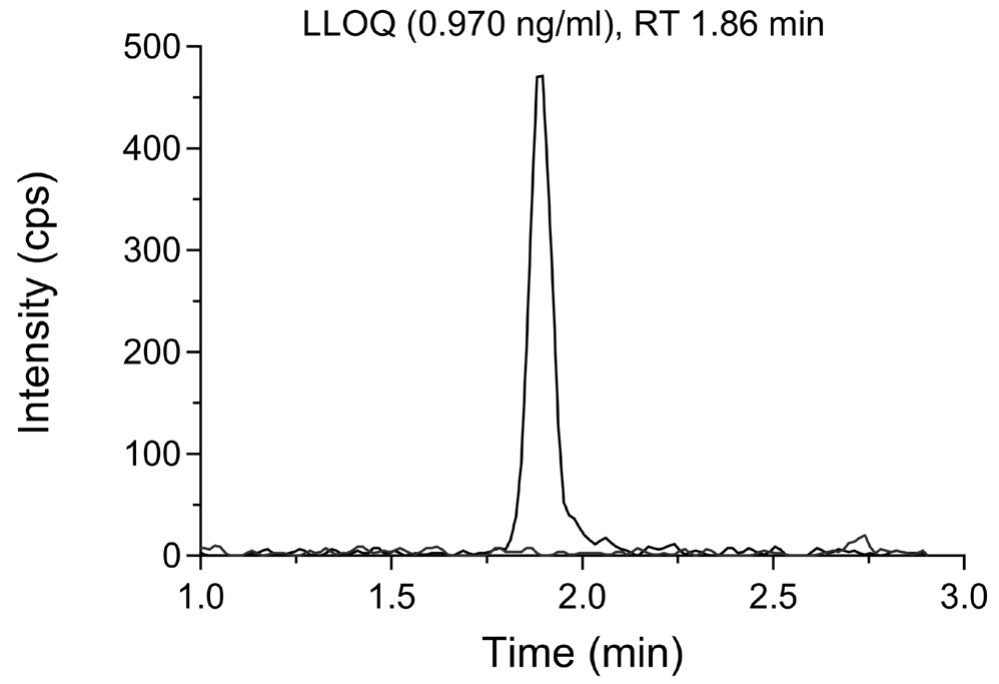
Extracted ion chromatogram of ivermectin in a whole blood sample at LLOQ concentration (0.970 ng/ml), overlaid with a blank sample.

A previous LC-MS method published by Duthaler *et al.* reported a 30% suppression effect in the plasma matrix, while whole blood had a 20% enhancement. However, the effect was similar between donors and the %CVs were low with small variations. In our case, there was no matrix effect found in the six sources of blank whole blood and plasma, and all sources were free from interfering peaks at the retention time of ivermectin. This is crucial to evaluate during method validation as it ensures that the analyte signal is not compromised by other components in the sample, thus ensuring the method's accuracy and reliability. The absence of interfering peaks at the retention time of ivermectin across all sources implies high specificity of the method. This ensures that the method can accurately distinguish ivermectin from other substances, which is essential for the accurate quantification of the drug in biological samples. Ivermectin-normalized matrix effect ratios (drug/stable isotope-labelled internal standard) were close to 1 with a low variation, suggesting minimal to no matrix effects ( Table 2) ( Bonfiglio *et al.*, 1999; Ismaiel *et al.*, 2008; Little *et al.*, 2006; Lynch, 2016; Matuszewski *et al.*, 2003; Taylor, 2005; Xia & Jemal, 2009; Ye & Pao, 2015; Zhou *et al.*, 2017). This indicates that the internal standard effectively compensated for any minor variations, enhancing the robustness of the method. EDTA and an alternative anticoagulant (i.e. Heparin, Table S3 ( Kaewkhao, 2023b)) in whole blood and plasma extractions including EDTA hemolysis plasma did not show any ion suppression or ion enhancement. Post-column infusion experiments showed no regions with severe matrix effects at or around the retention time of ivermectin, reinforcing the conclusion that the method is free from significant matrix interferences (Figure S10 and Figure S11 ( Kaewkhao, 2023b)). Four co-administered drugs were injected as neat solutions (100 ng/mL of lumefantrine, piperaquine, primaquine, and dihydroartemisinin) and did not show any interference with the quantification of ivermectin (Figure S12 ( Kaewkhao, 2023b)). This demonstrated method selectivity, ensuring that it can accurately measure ivermectin even in the presence of other co-administered drugs. The developed assays showed high sensitivity, resulting in clearly visible peak chromatograms at LLOQ (0.970 ng/ml) with over 10-fold signal-to-noise response ( Figure 4). The limit of detection (LOD) of ivermectin in whole blood and plasma samples was set to 0.485 ng/ml, resulting in a signal-to-noise ratio of more than 3.

**Table 2.** Absolute recovery, process efficiency and matrix effects of ivermectin in human CPD whole blood and plasma samples.

Matrix	Sample (concentration)	Absolute recovery ^*a*^ ± %CV	Process efficiency ^*b*^ ± %CV	Matrix factor ^*c*^	Normalized matrix factor ^*d*^
Whole blood	QC1 (3.39 ng/ml)	101 ± 6.03	104 ± 6.03	1.03	0.981
	QC3 (308 ng/ml)	96.3 ± 4.55	109 ± 4.55	1.13	1.00
	SIL QC1 (80 ng/ml)	116 ± 4.33	122 ± 4.33	1.05	
	SIL QC3 (80 ng/ml)	111 ± 4.18	123 ± 4.18	1.13	
Plasma	QC1 (3.39 ng/ml)	108 ± 4.82	107 ± 4.82	0.985	1.00
	QC3 (308 ng/ml)	117 ± 3.28	119 ± 3.28	1.02	1.00
	SIL QC1 (80 ng/ml)	105 ± 4.84	104 ± 4.84	0.986	
	SIL QC3 (80 ng/ml)	108 ± 4.14	110 ± 4.14	1.02	

All values are presented as the mean of five replicates of samples. ^*a*^ Absolute recovery = Peak response of the individual QC sample/Average peak response of post-spiked extracted blank sample. ^*b*^ Process efficiency = Peak response of the individual QC sample/Average peak response of reference solution. ^*c*^ Absolute matrix factor = Peak response of the post-spiked extracted blank sample/Average peak response of reference solution. ^*d*^ Normalized matrix factor (MF) = MF analyte/MF IS

The absolute recovery and process efficiency of ivermectin and its isotope-labelled internal standard (ivermectin-D2) in whole blood and plasma were in the range of 96–123% and 105–119%, respectively ( Table 2). However, the normalized matrix factor ratios for both low and high QC samples in whole blood and plasma samples were close to 1, demonstrating that the internal standard compensated fully for any variations seen in the recovery. Phospholipid removal was evaluated with various protein precipitation solutions during method development of whole blood sample. This technique was used as it is simple to apply and reduced the source of LC-MS/MS matrix effects (e.g., residual proteins or phospholipids) and therefore also resulted in the best recovery. Duthaler *et al.* reported similar recoveries in plasma and whole blood but they only used a simple protein precipitation that resulted in more matrix effects. Schulz *et al.* utilized a full solid phase extraction procedure and reported no matrix effects, but the recovery was lower at 80%. In summary, phospholipid removal offers significant advantages over protein precipitation, liquid-liquid extraction, and solid phase extraction in terms of simplicity, speed, and cost-effectiveness. Its targeted ability to reduce matrix effects and improve analyte recovery makes it an attractive choice for method development in plasma and whole blood sample analysis, particularly when high-throughput and time efficiency are critical. The benefit of phospholipid removal is the balance of simplicity and effectiveness, providing cleaner extracts with reduced matrix effects, leading to more accurate and reliable LC-MS/MS results.

There was no carry-over detected in triplicate injections of blank solution following the injection of five replicates of extracted ULOQ. Also, extracted blank whole blood and plasma samples did not show any significant carry-over peaks of ivermectin, or its stable isotope-labelled internal standard.

Schulz *et al.* reported poor freeze/thaw stability in whole blood, while plasma passed all stability tests. In our stability evaluation, ivermectin in whole blood and plasma were stable (± 5% deviation) during all stability tests, i.e., stability during five freeze/thaw cycles, short term stability at ambient temperature (22°C) and refrigerator (4°C) for 4 hrs, 24 hrs and 48 hrs, and pre-treated sample stability stored in refrigerator (4°C) for 24 hrs. Our results are in line with previous reports ( Chhonker *et al.*, 2018; Duthaler *et al.*, 2019). The LC autosampler stability test at 10°C, showed that extracted ivermectin whole blood samples were stable for at least 65 hrs before injection, and that extracted plasma samples were stable for at least 55 hrs. Overall, ivermectin is very stable in plasma and whole blood, and we have also shown that long-term stability of spiked stored samples for ivermectin quantification was stable in a deep freezer (-80°C) for at least 2 years and 9 months in plasma (whole blood not yet evaluated) (Table S4 ( Kaewkhao, 2023b)). No part of the developed method was particularly susceptible to stability issues, and this method should be straight forward to implement into a routine bioanalytical high-throughput laboratory.

### Clinical applicability and incurred sample reanalysis (ISR)

The validated method was implemented into our bioanalytical high-throughput laboratory and clinical whole blood and plasma samples were analyzed. The whole blood method was used to analyze 960 samples from an open-label study to evaluate the safety, tolerability, potential pharmacokinetic interactions and mosquito-lethal effects of orally administered ivermectin, primaquine, and dihydroartemisinin-piperaquine in healthy adult Thai subjects ( Kobylinski *et al.*, 2020). The plasma method was used to analyze 1,267 plasma samples from a phase II/III, randomized, placebo-controlled trial of the efficacy and safety of ivermectin in children and adult patients with dengue infection (NCT02045069), which will be published elsewhere. Here, we present the method performance of the QC data to demonstrate applicability during routine analysis of clinical trial samples. The selected calibration range for whole blood and plasma proved suitable for the concentration measurements of the study samples.

Three QC levels (low, medium and high) were analyzed with patient samples. One low QC sample out of fifty-one, and one of high QC sample out of fifty-four, were outside the ±15% accuracy during analysis of clinical patient whole blood and plasma samples, respectively. However, the variation in precision of all QC levels was less than 5% in both the whole blood and plasma analysis. To further evaluate the reliability and reproducibility of the validated methods, patient samples were randomly selected for incurred sample re-analysis (∼10% of the total study samples) across the concentration-time profile. The selected samples were analyzed in a separate run for incurred sample reanalysis (ISR) evaluation.

**Figure 5.**
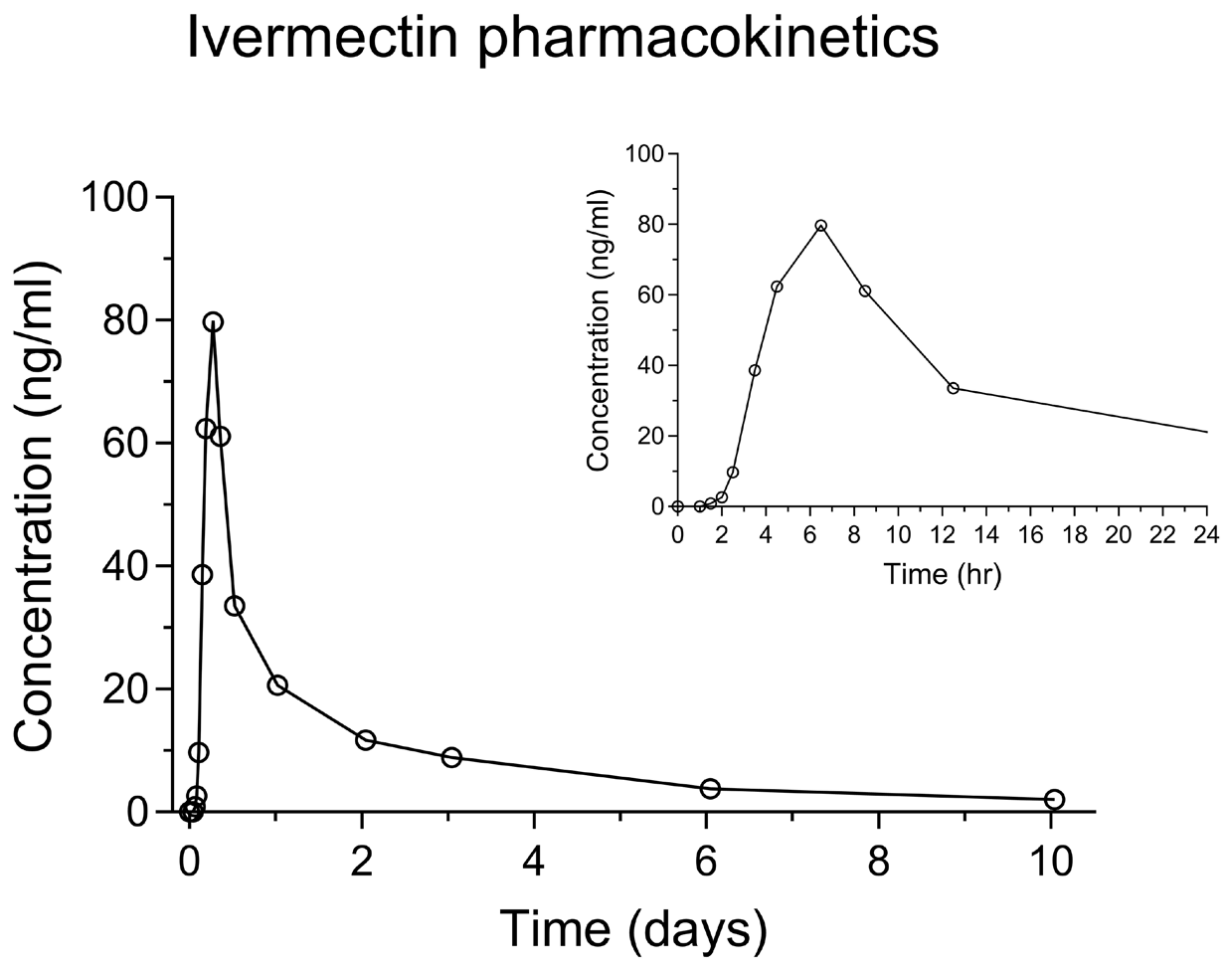
Whole blood concentration-time profiles of ivermectin after oral administration of a standard single dose (400 µg/kg) in one healthy Thai subject ( Kobylinski *et al.*, 2020). The inserted graph in the top right corner show the concentration-time profile during the first day after dosing.

The sample volume in the plasma study was very limited, and thus not enough to perform ISR. However, out of 54 ISR whole blood samples, over 90% of repeat samples met acceptance criteria, which is well above the pass requirement criteria of 67%. Below is a representative graph of a pharmacokinetic whole blood concentration-time profile in a single healthy Thai subject after receiving a single oral dose of ivermectin (400 µg/kg) ( Figure 5). Ivermectin administration resulted in peak concentrations of 36–119 ng/ml, similar to that previously described in patients and volunteers. Time to peak concentrations was determined to be 3–6.25 hours, and the lipophilic nature of ivermectin resulted in a large volume of distribution and a long terminal elimination half-life.

## Conclusions

In conclusion, we have developed and validated a LC–MS/MS method for the analysis of ivermectin in whole blood and plasma samples using the same extraction technique. The methods use a low sample volume of 100 µl and demonstrated a robust quantification of ivermectin over a range of clinically relevant concentrations related to therapy. The use of Hybrid SPE ^®^Plus extraction plates (phospholipid removal) to achieve a simple sample extraction technique with a high recovery. The Hybrid SPE ®Plus extraction proved to effectively adsorb blood components such as hemoglobin from whole blood samples without the loss of ivermectin. This technique was also easy to automate using a liquid handler for high-throughput analysis of clinical whole blood and plasma samples. The validated high-throughput method demonstrated to be reliable and reproducible when implemented in our bioanalytical clinical laboratory, and could enable us to elucidate the pharmacokinetic and pharmacodynamic relationships of ivermectin when used for its many indications.

## Data Availability

Figshare: Data analysis of ivermectin plasma and whole blood method validation. https://doi.org/10.6084/m9.figshare.24647718 ( Kaewkhao, 2023a). This project contains the following underlying data: Batch 2 plasma.txt (Quantification of raw data of day 1, for the accuracy and precision used in ANOVA calculations) Batch 3 plasma.txt (Quantification of raw data of day 2, for the accuracy and precision used in ANOVA calculations) Batch 4 plasma.txt (Quantification of raw data of day 3, for the accuracy and precision used in ANOVA calculations) Batch 5 plasma.txt (Quantification of raw data of day 4, for the accuracy and precision used in ANOVA calculations) Batch 2 WB.txt (Quantification of raw data of day 1, for the accuracy and precision used in ANOVA calculations) Batch 3 WB.txt (Quantification of raw data of day 2, for the accuracy and precision used in ANOVA calculations) Batch 4 WB.txt (Quantification of raw data of day 3, for the accuracy and precision used in ANOVA calculations) Batch 5 WB.txt (Quantification of raw data of day 4, for the accuracy and precision used in ANOVA calculations) Batch 1 plasma Recovery.txt (Peak areas of extracted QC samples, blank plasma post spiked and reference (RS) in neat solution for recovery and matrix effect calculations) Batch 1 Recovery WB.txt (Peak areas of extracted QC samples, blank whole blood post spiked and reference (RS) in neat solution for recovery and matrix effect calculations) Batch 6 plasma.txt (Quantification data stability testing, Freeze/thaw cycle 3 and cycle 5 and Pre-treated 24 hrs in plasma sample) Batch 7 plasma.txt (Quantification data stability testing, Short term stability for 4 and 48 hrs in RT, 48 hrs in 4 °C in plasma sample) Batch 6 WB.txt (Quantification data stability testing, Freeze/thaw cycle 3 and cycle 5 in whole blood sample) Batch 7 WB.txt (Quantification data stability testing, Short term stability for 4 and 48 hrs in RT, 48 hrs in 4 °C and Pre-treated 24 hrs. in whole blood sample) Batch 3 plasma Auto stab.txt (Quantification data autosampler stability testing, comparing the difference in quantified concentration from original injected samples re-injection 55 h later) Batch 3 WB Auto stab.txt (Quantification data autosampler stability testing, comparing the difference in quantified concentration from original injected samples re-injection 55 h later) Figshare: Supplementary of ivermectin plasma and whole blood method. https://doi.org/10.6084/m9.figshare.24647943 ( Kaewkhao, 2023b). This project contains the following extended data: Supplementary.pdf (Supplementary material) Data are available under the terms of the Creative Commons Attribution 4.0 International license (CC-BY 4.0).
