# Supplementary material for: Determination of ivermectin in plasma and whole blood using LC-MS/MS
Source: Wellcome Open Res. 2024 Aug 5;9:231. Originally published 2024 Apr 24. [Version 2] doi: 10.12688/wellcomeopenres.20613.2 (PMC11443190; doi:10.12688/wellcomeopenres.20613.2)
Supplement: Peer review history

# Reviewer report: Reviewer response for version 2

**DOI:** 10.21956/wellcomeopenres.25164.r98725

**Published:** 2024-09-30

**Authors:** Vieyle Cortez

**Affiliations:** 1 Universidad de Alicante, Alicante, Spain

I have reviewed the manuscript entitled “Determination of ivermectin in plasma and wholeblood using LC-MS/MS". The manuscript describes the validation of an analytical method for the determinations and quantification of ivermectin in human's plasma and whole blood using LC-MS/MS. In general, the methodology is adequately described and validated according to the international guidance's.

**I have the following comments:**

 1. In the abstract highlight the novelty of this method, which I think is the use of an effective automated sample extraction  using a hybrid-solid phase technique for the extraction of ivermectin from blood and plasma. Also, specify that human samples are involved.

 2. In Table 2, it is not clear what is meant by the acronym SIL.

 3.In the results and discussion section (Method development), I suggest move the sentence "Hypersil gold C18 100×2.1mm 5μm (Thermo Scientific, MA, USA, 25005-102130), Gemini C18 50×2.0mm 5μm (Phenomenex, CA, USA, 00B-4435-B0), Zorbax SB-CN 50×4.6mm 3.5μm (835975-905) and Poroshell EC C18 50×3 mm 2.7 μm (699975-302) (Agilent technologies, CA, USA)" to the Method section. This is because nothing in the Methods refers to evaluating four reversed-phase columns to optimise the LC technique.

Is the rationale for developing the new method (or application) clearly explained?

Yes

Is the description of the method technically sound?

Yes

Are the conclusions about the method and its performance adequately supported by the findings presented in the article?

Yes

If any results are presented, are all the source data underlying the results available to ensure full reproducibility?

Yes

Are sufficient details provided to allow replication of the method development and its use by others?

Yes

Reviewer Expertise:

Chemical  ecology, analysis of ivermectin in biological samples

I confirm that I have read this submission and believe that I have an appropriate level of expertise to confirm that it is of an acceptable scientific standard.

# Reviewer report: Reviewer response for version 2

**DOI:** 10.21956/wellcomeopenres.25164.r92169

**Published:** 2024-09-21

**Authors:** Helgi Jung-Cook

**Affiliations:** 1 Universidad Nacional Autónoma de México, Mexico City, USA

The authors responded satisfactorily to the questions and made the requested changes. I have no further comments.

Is the rationale for developing the new method (or application) clearly explained?

Partly

Is the description of the method technically sound?

Yes

Are the conclusions about the method and its performance adequately supported by the findings presented in the article?

Yes

If any results are presented, are all the source data underlying the results available to ensure full reproducibility?

Yes

Are sufficient details provided to allow replication of the method development and its use by others?

Yes

Reviewer Expertise:

Pharmacokinetics

I confirm that I have read this submission and believe that I have an appropriate level of expertise to confirm that it is of an acceptable scientific standard.

# Reviewer report: Reviewer response for version 1

**DOI:** 10.21956/wellcomeopenres.22816.r85074

**Published:** 2024-06-21

**Authors:** Helgi Jung-Cook

**Affiliations:** 1 Universidad Nacional Autónoma de México, Mexico City, USA

Considering that there are different bioanalytical methods previously published for the quantification of ivermectin in plasma, the novelty of the method is the use of a hybrid-solid phase technique for the extraction of ivermectin from blood and plasma. The analytical assay was fully validated according to the international guidance’s.

 I have the following comments:

1. Authors state that ivermectin is the most widely used anthelmintic drug worldwide.  References should be added to support this assertion, since according to the clinical guidance’s, benzimidazoles are the most widely prescribed anthelmintic drugs.  The WHO also has classified this drug as an antifilarial drug.
2. Considering that ivermectin is highly bound to plasma proteins (albumin), it would be expected that the diffusion to red blood cells should low.  Please explain why it was decided to validate the method using both plasma and red blood cells.
3. For the incurred sample reanalysis authors used plasma samples from a clinical study performed in malaria patients, previously reported by Kobylinsky et al. Authors state that ivermectin is very stable, however the previous study was published in 2019.  These results could only be included if the authors can document the stability of the samples from the time they were analyzed in the first study to the time of analysis in the second study and thus support the reliability of the data.
4. It would be convenient to include a table with the inter and intra-day precision and accuracy results.

Is the rationale for developing the new method (or application) clearly explained?

Partly

Is the description of the method technically sound?

Yes

Are the conclusions about the method and its performance adequately supported by the findings presented in the article?

Yes

If any results are presented, are all the source data underlying the results available to ensure full reproducibility?

Yes

Are sufficient details provided to allow replication of the method development and its use by others?

Yes

Reviewer Expertise:

Pharmacokinetics

I confirm that I have read this submission and believe that I have an appropriate level of expertise to confirm that it is of an acceptable scientific standard, however I have significant reservations, as outlined above.

# Response

**Published:** 2024-07-18

**Authors:** Natpapat Kaewkhao

**Affiliations:** Pharmacology, Mahidol Oxford Tropical Research Unit, Bangkok, Bangkok, Thailand

Reviewer 2: Comments

**1.    Authors state that ivermectin is the most widely used anthelmintic drug worldwide.  References should be added to support this assertion, since according to the clinical guidance’s, benzimidazoles are the most widely prescribed anthelmintic drugs. The WHO also has classified this drug as an antifilarial drug.**

 Thank you for this clarification. We have revised the abstract and introduction accordingly. The WHO List of Essential Medicines (2023) lists Ivermectin under Anthelmintics, with two subcategories; Intestinal anti-helminthic and Anti-filarial drug. Thus, ivermectin is classified as both an anti-helminthic drug, broadly treating infections caused by parasitic worms (helminths), and specifically as an anti-filarial drug, effective against filarial infections caused by specific types of parasitic worms known as filariae.  We have addressed this point in the revised version in “Abstract” and “Introduction”

**2.    Considering that ivermectin is highly bound to plasma proteins (albumin), it would be expected that the diffusion to red blood cells should low.  Please explain why it was decided to validate the method using both plasma and red blood cells.**

 The reviewer is correct, ivermectin do not accumulate in red blood cells, and is found at higher concentrations in plasma compared to whole blood. The reason for developing both plasma and whole blood methods was that the laboratory was asked to measure ivermectin in two separate clinical trials. One collected plasma samples while the other study collected whole blood samples. Additionally, depending on the aim of the study, whole blood or plasma would be the preferred matrix. For example, when evaluating the mosquito-lethal effects of ivermectin during a mosquito blood meal, whole blood pharmacokinetics would be most appropriate. We also added text and a reference in the end of the discussion section, referencing the distribution of ivermectin into blood and plasma.

**3.    For the incurred sample reanalysis authors used plasma samples from a clinical study performed in malaria patients, previously reported by Kobylinsky et al. Authors state that ivermectin is very stable, however the previous study was published in 2019. These results could only be included if the authors can document the stability of the samples from the time they were analyzed in the first study to the time of analysis in the second study and thus support the reliability of the data.**

 We thank the reviewer for highlighting this and we agree that this was unclear. The stability tests were conducted on spiked stored samples, and to evaluate long-term stability, we use freshly spiked plasma or whole blood calibrators to assess the stability of old stored sample sets. We have revised the text in the “Stability result” section. Furthermore, the performance of the plasma samples reported here was indeed analyzed at the time when the main clinical trial was conducted and reported.

**4.    It would be convenient to include a table with the inter and intra-day precision and accuracy results.**

 Thank you for this suggestion. We have added this data in a graphical format (Figure 2 and Figure 3), which is described in “Results & discussion: Method validation”. The figure includes error bars that illustrate the %RSD, thereby conveying the same information as a table would, but in a more intuitive and visual manner. We believe this approach enhances the clarity and accessibility of the data for readers.

# Reviewer report: Reviewer response for version 1

**DOI:** 10.21956/wellcomeopenres.22816.r85067

**Published:** 2024-06-20

**Authors:** Daryl J. Murry, Mamunur Rashid

**Affiliations:** 1 Pharmacy Practice and Science, University of Nebraska Medical Center, Omaha, Nebraska, USA

I have reviewed the manuscript entitled “ **Determination of ivermectin in plasma and whole blood using LC-MS/MS.”**The manuscript describes the validation of a bioanalytical method for the quantitation of ivermectin in plasma and whole blood using LC-MS/MS. Overall, the manuscript is interesting, and the methodology is adequately described.

 Major comments

1. The advantage of this method over existing approaches should be further discussed.
2. Why did the author estimate Ivermectin in plasma if ivermectin is accumulates in red blood cells, as the author discussed in the conclusion?
3. It is unclear why the QC experiment was conducted at 4.64 (low), 56.4 (medium), and 524.0 (high) ng/mL, whereas the upper limit of quantification of calibration is 384 ng/mL?
4. Why did the author suddenly switch the mobile phase from 100 % to 0 % within 1 minute in the LC-MS/MS gradient system? Is one minute enough for the column to separate the analytes? There is no gradient in this LC method. Please describe the gradient and time needed to re-equilibrate the bioanalytical column.
5. How did the author process the whole blood samples after freezing them at -80°C? explain briefly in the manuscript. Does blood become normal, or is there any clumping or lysis after freezing at -80°C?
6. The inclusion of a RBC portioning study to evaluate the Ivermectin partitioning between RBC and plasma should be included
7. It is recommended that the author also include the plasma concentration versus time profile in the manuscript. 

Is the rationale for developing the new method (or application) clearly explained?

Yes

Is the description of the method technically sound?

Yes

Are the conclusions about the method and its performance adequately supported by the findings presented in the article?

Partly

If any results are presented, are all the source data underlying the results available to ensure full reproducibility?

No

Are sufficient details provided to allow replication of the method development and its use by others?

Yes

Reviewer Expertise:

bioanalytical, pk/pd

We confirm that we have read this submission and believe that we have an appropriate level of expertise to confirm that it is of an acceptable scientific standard, however we have significant reservations, as outlined above.

# Response

**Published:** 2024-07-18

**Authors:** Natpapat Kaewkhao

**Affiliations:** Pharmacology, Mahidol Oxford Tropical Research Unit, Bangkok, Bangkok, Thailand

**Reviewer 1: Comments**

**1. The advantage of this method over existing approaches should be further discussed.**

 I have addressed this point in the revised version by adding a more detailed discussion on the advantages of this method over existing approaches in the section of “Results & discussion: Method Validation” at paragraph 3 and 4.

**2. Why did the author estimate Ivermectin in plasma if ivermectin is accumulates in red blood cells, as the author discussed in the conclusion?**

 Apologies for this misunderstanding. Actually, ivermectin do not accumulate in red blood cells, and is found at higher concentrations in plasma compared to whole blood. We have adjusted the text in “Conclusions” to clarify the statement about hemoglobin-binding to the extraction plate.

 “The Hybrid SPE ®Plus extraction proved to effectively adsorb blood components such as hemoglobin from whole blood samples without the loss of ivermectin.” We also added text and a reference in the end of the discussion section, referencing the distribution of ivermectin into blood and plasma.

**3. It is unclear why the QC experiment was conducted at 4.64 (low), 56.4 (medium), and 524.0 (high) ng/mL, whereas the upper limit of quantification of calibration is 384 ng/mL?**

 We thank the reviewer for discovering this typo in the submitted document. The correct concentrations of three QC levels has been specified in the revised version.

**4. Why did the author suddenly switch the mobile phase from 100 % to 0 % within 1 minute in the LC-MS/MS gradient system? Is one minute enough for the column to separate the analytes? There is no gradient in this LC method. Please describe the gradient and time needed to re-equilibrate the bioanalytical column.**

 The elution of ivermectin is performed under isocratic conditions (mobile phase A) during the first 2.1 minutes. However, a washout step (mobile phase B, 2.1-3.1 min) was also implemented to remove any residual compounds. The program then return to isocratic conditions using only mobile phase A for the remaining time (3.1 – 5.0 min), which is enough to equilibrate the column before the next injection.  

**5. How did the author process the whole blood samples after freezing them at -80°C? explain briefly in the manuscript. Does blood become normal, or is there any clumping or lysis after freezing at -80°C?**

 We have addressed this point in the revised version by adding a more detailed description in section “Methods: Sample preparation”.

**6. The inclusion of a RBC portioning study to evaluate the Ivermectin partitioning between RBC and plasma should be included.**

 As the clinical studies were conducted as a collaboration, we did not have direct input in the planning or execution of the clinical studies. Also, one study collected plasma samples while the other study collected whole blood, but none of them collected both. However, we have added information from other publications that explore the differences between ivermectin concentrations in plasma and whole blood.

**7. It is recommended that the author also include the plasma concentration versus time profile in the manuscript.**

 We would like to comply with the reviewer’s request. However, the pharmacokinetic evaluation will be published elsewhere, within the main study paper. Instead, we have chosen to present the method performance of the QC data as this demonstrates the appropriateness of the developed assay for the intended purpose. We have also added this information to the “Clinical Applicability” section in the “Results and Discussion”.
